# Investigating the Effects of Ibuprofen on the Gene Expression Profile in Hippocampus of Mice Model of Alzheimer’s Disease through Bioinformatics Analysis

**DOI:** 10.22037/ijpr.2019.15485.13125

**Published:** 2020

**Authors:** Mona Zamanian-Azodi, Mostafa Rezaei-Tavirani, Majid Rezaei-Tavirani

**Affiliations:** a *Proteomics Research Center, Shahid Beheshti University of Medical Sciences, Tehran, Iran. *; b *Proteomics Research Center, Faculty of Paramedical Sciences, Shahid Beheshti University of Medical Sciences, Tehran, Iran. *; c *Faculty of Medicine, Iran University of Medical Sciences, Tehran, Iran.*

**Keywords:** Non-steroidal anti-inflammatory drugs, Hippocampus, Alzheimer’s disease, Transcriptome, Protein-protein interaction network analysis, Penk

## Abstract

Non-steroidal anti-inflammatory drugs (NSAIDs) are identified as effective in many diseases. One of which is neurodegenerative diseases including Alzheimer disease (AD). In this study gross alteration of gene expression in AD mice by ibuprofen treatment is investigated via Protein-protein interaction network (PPI) analysis. Expression profiling of microarray dataset GSE67306 was retrieved from GEO database and analyzed via GEO2R tool. PPI analysis was performed via Cytoscape 3.7.0. and its plug-ins including Network Analyzer, Gene MANIA, and CluePedia. Numbers of 10 central genes including Htr1a, Sstr2, Drd2, Htr1b, Penk, Pomc, Oprm1, Npy, Sst, and Chrm2 were identified as potential biomarkers. However, the role of Penk gene was highlighted. The finding indicates that ibuprofen changes gene expression level of several genes that are involved in AD.

## Introduction

Within aging population in the world, the number of dementia patients particularly Alzheimer disease (AD) is increasing (1). The chief pathological molecule in AD is amyloid β-peptide (Aβ) (1). On the other hand, non-steroidal anti-inflammatory drugs (NSAID) as firstly introduced in 1960s, was broadly used drug worldly. It was estimated that the annual application of this medicine is over 100 million in the U.S. The benefits of NSAIDs is extensive in medical fields, in which can be applied as pain reliever, and inflammation reducer by inhibiting the prostaglandins (2). The diseases that were previously examined with NSAIDs include sepsis (3), cancer, (4), Parkinson disease (5), and Alzheimer’s disease (6). In each of these diseases, the mechanisms of NSAIDs action have been described as follow: the levels of prostacyclin and thromboxane that cause fever, tachycardia, oxygen consumption, and lactic acidosis in patients with sepsis reduces as treated with ibuprofen (3). NSAIDs in addition, by inhibiting cox-2 hinder the development of many cancer types and therefore, show properties (4). Neuroprotective role of this drug family especially ibuprofen (IBU) was identified as helpful in reducing neuroinflammation (5). In general, ibuprofen is a nonsteroidal anti-inflammatory drug effective on prostaglandin-synthase accounts for as one of the most frequent useful fast pain reliever (7, 8). The approval of this safe drug which could be used for both short-term and long-term treatments was in 1968 in England (9). In addition, NSAIDs such as ibuprofen claimed to have some positive effects on memory lose in mice with Alzheimer’ disease (AD) through molecular alterations (6, 10). They act as inhibitors of two isoforms COX-1 and COX-2, the cyclooxygenase enzymes that are involved in AD and are parts of innate immune system producing prostaglandins. Both the two immunoregulators (COX-1 and COX-2) of brain are appropriate targets for neuroinflammation treatments (11). This is the famous mechanism by which NASIDs are known to be effective (6). Inhibition of COXs production lead to reduction of Aβ concentration and therefore its impact as the memory decliner hinders (11). On the other hand, systematic molecular assessment could be beneficial in this sense. In this regard a set of molecules including genes, small RNAs, and proteins could be recognized through high-throughput methods as biomarkers (12). Gene expression microarray analysis could be helpful in this regard to provide molecular nature understanding of the designated disease (13). Likewise, pharmacological properties of some drugs could be clearer by large-scale molecular investigations (14). Here, the gene expression profile of AD in the treated mice with ibuprofen (dosage of 375 ppm) is investigated via protein interactions map to facilitate uncovering of ibuprofen effect on gene expression profile of AD mice.

## Experimental

The expression data GEO series accession number= GSE67306 via platform, GPL6246 was obtained from the Gene Expression Omnibus (GEO, http://www.ncbi.nlm.nih.gov/geo/) (13). The conducted study was entitled “Effect of ibuprofen on hippocampal gene expression in APP-PS1 mice”. The finding was Public on Jun 14, 2016 in GEO database. This study was aimed to identify the preventive effects of NSAID on Mus musculus (Mouse) AD model. There were 19 samples belong to groups of control (wild type and AD type) and NSAID treatment groups including wild type and AD model. In our investigation, two sample groups were defined as AD male mice chow without additive and AD male mice with ibuprofen chow with the dosage of 375 ppm. 

Here, the accession numbers of each samples (SAN) applied in our study are as follows

For each groups, four samples were designated and the dataset was analyzed and compared via the web-based tool GEO2R (http://www.ncbi.nlm.nih.gov/geo/info/geo2r.html). Before the *t*-test, box plotting was used for cross comparison between groups of interest. The median-centered data implies that these groups of samples are comparable in terms of expression profile. The top 250 differentially expressed genes (*p*.value ≤ 0.05) are assigned by GEO2R and the most significant differential expressed genes are selected. At first, the genes without names were omitted from the dataset and the remaining ones were considered for more analysis. Fold change above 1.4 was considered to determine most significant DEGs which distinct the treated AD mice from the untreated ones. After this evaluation, these genes were included in protein-protein interaction network analysis. The protein-protein interaction network was obtained from STRING database (https://string-db.org/) and visualized in Cytoscape platform version 3.7.0. (https://cytoscape.org/) (15, 16). After network construction, network statistics was obtained by Network Analyzer by identifying the topological features. Degree and betweenness centrality were considered as network centrality characteristic (17). For better filtering the nodes with the highest values of centrality, hub-bottleneck was assigned for those with highest values of degree, betweenness, and closeness. These nodes were then pursued for the possible association types via GeneMANIA. In this regard a sub-network of them was constructed (12) and the source of co-expression data is from different studies. After identification of central nodes, they were then queried for the action type linkage and their related miRNAs as a constructed map in *Cytoscape *and its plug-in CluePedia (CluePedia miRanda-miRNAs V5-2012-87-19.txt.gz file and mirecords.umn.edu.validated.miRNAs.2010-11-25.txt.gz). For both predicted and validated interactions, the score cut off was set to default option 0.6 (18). The action type analysis was carried out simultaneously in the same platform and actions of activation, inhibition, expression, and post translation modifications were searched for the designated nodes by considering the kappa score cut off = 0.6. The range for kappa score is from 0 to 1. Moreover, STRING Action File in CluePedia Panel was the source for action identification. 

## Results

**Figure1:**
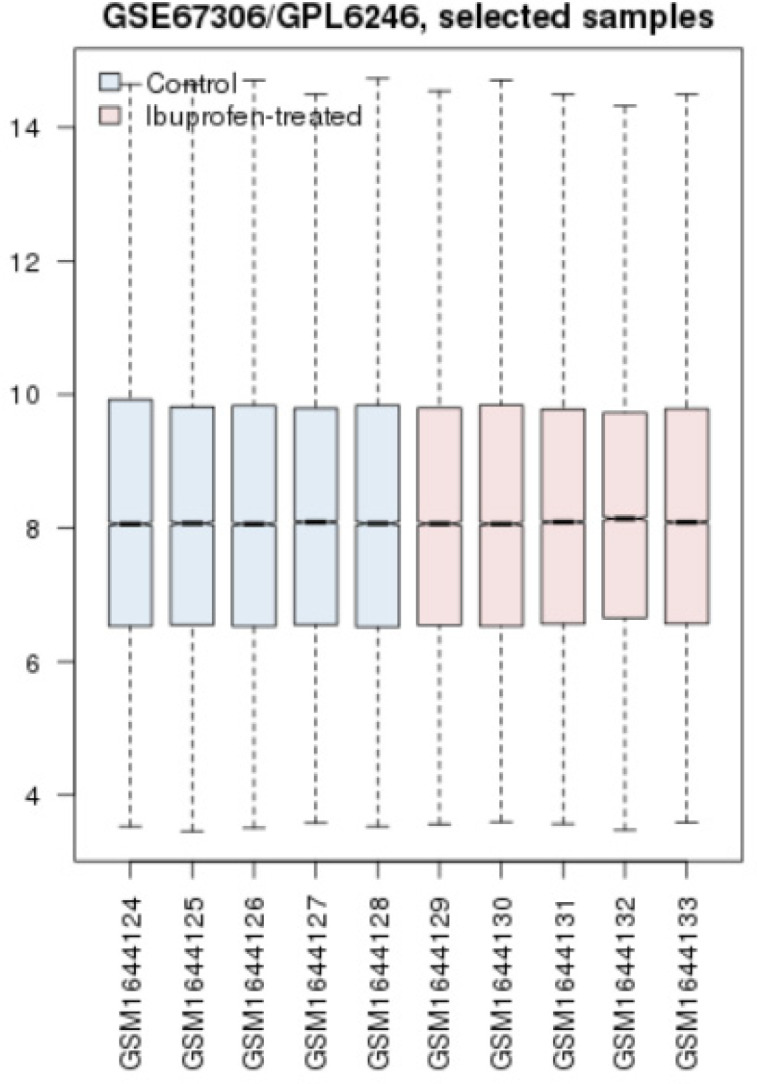
Cross comparison of two groups of AD mice and AD mice treaded with ibuprofen. The blue boxes refer to controls and the pink ones refer to AD-treated samples

Prior to statistical analysis of differential expressed genes in sample comparison, another statistical method was applied. A cross comparison was handled to evaluate whether the samples are comparable in terms of expression difference. Box plotting was the method to carry out this investigation as indicated in Figure 1. After this, top 250 significant DEGs in the AD mice treated with ibuprofen were obtained by GEO2R analysis. The genes that were without symbols were removed from our analysis. From top 250 significant DEGs, 136 ones were with gene names. Among these 136 genes, five genes were identified as the most significant DEGs. The numbers of four most significant DEGs also were determined among the DEGs below rank of 250 significant individuals. All together nine genes were gathered (see table 2). 

Cross comparison expressed that the sample data are median-centered in Figure 1 and therefore qualified for further investigations in terms of expression comparison.

**Figure 2:**
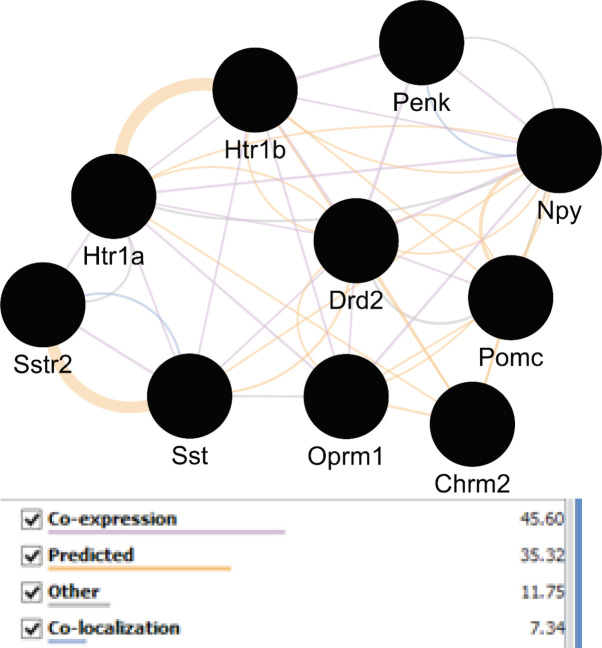
Network view of central elements of the main network corresponding to each other via different relationships. Different edges colors implies on different associations between these nodes

A network was constructed from the nine genes of table 1 plus 100 related neighbor nodes. Snord115 was not recognized by STRING and four genes including DSP, Ecel1, zcchc5, and Gm7120 were isolated nodes. The main connected component includes 104 nodes and 2520 edges. Top 10% of nodes based on three centrality parameters including degree, betweenness, and closeness values were selected to determine central nodes. In this regard, the numbers of 11 genes were identified. The genes that were common in at least two categories were selected as central nodes. As it is shown in the table 3, ten genes are introduced as central genes. To visualize the central nodes linkage, GeneMANIA was used to declare these associations from different aspects (See figure 2).

**Figure 3:**
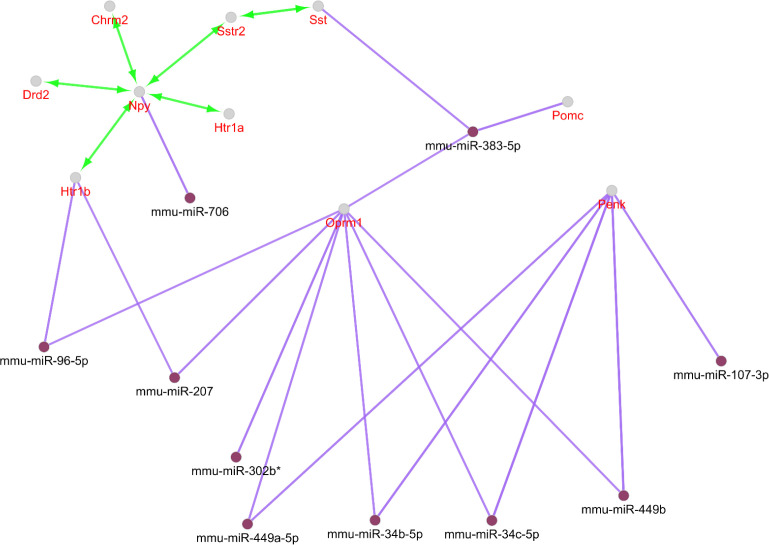
Action view and regulatory network for the central nodes of the original network. The score cut off for miRNA interactions is set to 0.6. A cut off 10 miRNAs are set for target central nodes. The actions include activation (green), expression (yellow), and inhibition (red). However, the last two are not concluded in any result. The edge thickness indicates the max and min scores (cut off for these action scores was set to 0.5 (medium)). * refers to the maximum kappa score value (0.796).

Action type and miRNAs relation to the central nodes analysis can provide more knowledge about different scales of these elements of the network strength. Activation is the only present action among our nodes considering the designated cut off. Seven genes among the ten ones are in this type action relation. Four groups of miRNAs targets can be realized from the interactions. These include Penk and Opmr1 as the common targets of four miRNAs. Pomc and Sst are both targets of miR-383-5p. Opmr1 and Htr1b are also targeted by miR-207. Pomc and Opmr1 in addition are common for miR-383-5p. It should be noted that miR-383-5p has two groups of targets (See Figure 3). 

**Figure 4:**
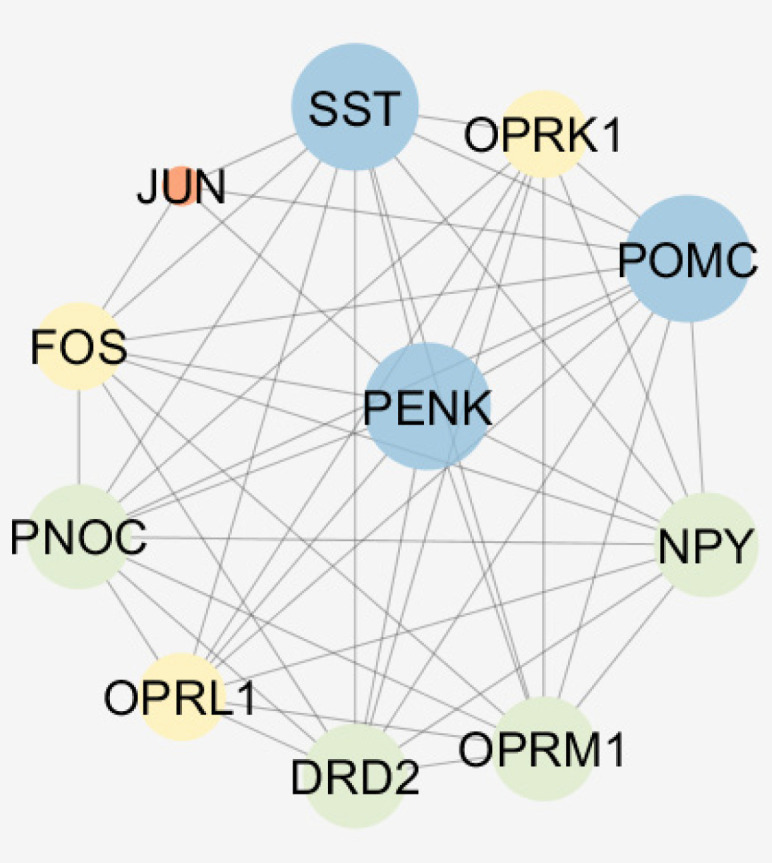
A sub-network including PENK and 10 relevant genes. The nodes are layout based on degree value. Bigger size and blue color refer to more connections. STRING application of Cytoscape is used to construct the sub network

**Figure 5:**
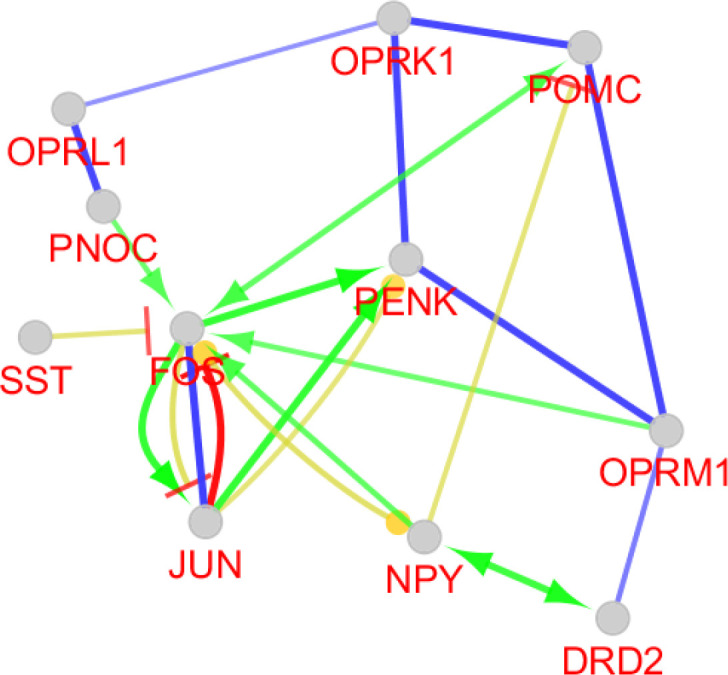
Action map including activation (green), inhibition (red) expression (yellow), and binding (blue) for PENK and its 10 relevant genes is presented. CluePedia application of Cytoscape is used to construct the action map

The results indicate that PENK (as a query gene) plays a crucial role in the network and also among the differentially expressed genes so in connections between this gen and numbers of 10 relevant genes were visualized (see Figure 4). It may be suggested that Oprm1 as like PENK is a crucial gene due to its important connections but it is not a query gene. Action map (Figure 5) revealed more details about these relationships.

AD is known to be one of the heterogeneous neurodegenerative diseases that is not well recognized (1, 19). NSAIDs, an anti-inflammatory drug family has been shown to have some effects on neurodegenerative disorders such as Alzheimer disease. The mechanisms associated are under investigation and one of which is suggested to be the contribution of COX genes. To explore further in this regard and to gain a better knowledge of the molecular events after treatment with ibuprofen in AD mice, in the present study, a bioinformatics approach based on network analysis is conducted. In this regard, the DEGs are identified using GEO2R, an analytical web-based tool in GEO Database. At first, the expression data from two groups were screened for data quality for comparison purposes. As it is clear from figure 1, the data are median-centered and qualified to be compared. The genes from this comparison were explored for significant altered expressions. In Table 2, 9 genes are presented as the most significant DEGs in AD mice exposed to ibuprofen related to the untreated ones. The data indicated that Tdo2 has the highest fold change (2.5) while Eif2s3y and snord115 show the lowest fold changes (1.4). Of these elements, all are down-regulated except Penk, Ecel, and Snord115. Protein-protein interaction network used to find interaction properties of these nine genes. The query from STRING plug-in identified no direct interactions between these genes (the data is not shown). Adding 100 neighbor genes to the main 9 genes implies that most of these genes are in interaction; however, not directly. 

As highlighted before, Tdo2 is the most down-regulated gene in the presence of ibuprofen treatment. Its high expression in AD has correlation with cox gene activations that could result in mental abnormalities (6). The network prediction of ibuprofen influence on AD mice was then conducted by using DEGs. The statistical analysis for this constructed was carried out by Cytoscape plug-in, Network Analyzer. A total of 10 nodes were concluded after data processing and identification of common genes between the highest values of centrality parameters. Among the top central nodes, only Penk is from the significant altered expressed genes group in the treated subjects. The rest of genes are from neighbor ones that were added to the query genes. The three criteria for analyzing centrality in this network were assigned as degree, betweenness, and closeness. 

It can inferred that these genes are close in degree and closeness centrality amounts but not in betweenness scores. The ten central nodes can be introduced as hubs. Penk which is ranked as the fifth gene based on degree value, is characterized with high value of CC. there is no proper value of betweenness for Penk. This node as indicated before is from the query DEGs and could have additional values.

 Further analysis revealed that top amount of BC (0.070) belong to Eif2s3y (see Table 3). However, due to low value of degree it is not included in this table as central nodes. Eif2s3y could be considered as a fit bottleneck in the network stability. It is the fourth ranked bottleneck in the network after Chrm2, Eif2b1, and Htr1a. 

Following identification of central nodes, the annotation study of these agents was performed and neither mutual biological processes nor pathways were documented for them. Nevertheless, GeneMANIA identified the important relation between the central nodes of the original network in Figure 2. It is clear that central genes are mainly corresponded based on co-expression. Furthermore, other aspects of relations between these agents were also searched and CluePedia explored some of them. In this analysis as it is indicated in figure 3, Penk and Oprm1 are the chief targets for a number of microRNAs. 

A literature review of some of the central genes can provide more information regarding the findings. Penk is a gene that proved to have significant differential expression (up-regulation) in the treated subject. Additionally, this gene also displays centrality properties in the network of DEGs. Reduction of mRNA expression of this gene has been previously reported by many studies for AD (6, 20). Expression change of preproenkephalin in Parkinson disease also is reported (21). As it is shown in figure 3, Penk is targeted by five microRNAs, in which four of them are common with oprm1. Up-regulation of Penk by ibuprofen in AD is a critical point which has effects on the interactome integrity and control. As it is depicted in Figure3, Oprm 1 not only is targeted by four common microRNAs with Penk but also has common microRNAs with Pomc, Sst, and Htr 1b. Direct co-expression relationships such as Penk - Htr 1b, Htr 1b – Sst, and Sst – Oprm 1 which are shown in Figure 2, are corresponded to the excellent role of Penk in combination with the other central nodes such as Htr 1b, Sst, and Oprm 1 in effect of ibuprofen in AD. 

Critical role of PENK considering expression change and centrality parameters required more investigation about its characterization. Therefore, the numbers of 10 relevant genes were identified which are connected to PENK (see figure 4). Action map is used as a screen toll to determine more important ones that can regulate PENK. As it is clear in Figure 5, JUN and FOS are the two direct activators of PENK. Based on Figures 3 and 5, NPY is the other important gene that plays significant role in action maps. Five activation actions are issued from NPY which has effect indirectly on PENK via Htr1b. Previous studies demonstrtaed that both JUN and FOS are two significant players in AD (22). These finding are consistent with direct relationship between PENK and JUN and FOS genes. However, these two related genes were not included in the 250 query significant genes.

**Table 1:** The list of samples underlying investigation in this study

**GEO SAN**	**Samples**
GSM1644124	Hippocampus_APPS_Con_rep1
GSM1644125	Hippocampus_APPS_Con_rep2
GSM1644126	Hippocampus_APPS_Con_rep3
GSM1644127	Hippocampus_APPS_Con_rep4
GSM1644128	Hippocampus_APPS_Con_rep5
GSM1644129	Hippocampus_APPS_Ibu_rep1
GSM1644130	Hippocampus_APPS_Ibu_rep2
GSM1644131	Hippocampus_APPS_Ibu_rep3
GSM1644132	Hippocampus_APPS_Ibu_rep4
GSM1644133	Hippocampus_APPS_Ibu_rep5

**Table 2:** The most significant DEGs scores in the treated AD mice with ibuprofen relative to the AD mice. Three are up-regulated while six are down-regulated. Tdo2 is the most down-regulated gene

**Row**	**Gene Name**	**FC**	***P*** **-value**	**Regulation**
1	Penk	1.7	≤ 0.0007	Up
2	Dsp	1.9	≤ 0.0010	Down
3	Ecel1	1.6	≤ 0.0010	Up
4	Il1rl	1.5	≤ 0.0020	Down
5	Tdo2	2.5	≤ 0.0030	Down
6	zcchc5	1.6	≤ 0.0040	Down
7	Gm7120	1.7	≤ 0.0070	Down
8	snord115	1.4	≤ 0.0100	Up
9	Eif2s3y	1.4	≤ 0.0300	Down

**Table 3:** The list of central genes with high centrality values. Top 10% of genes based on degree, betweenness and closeness were considered and the genes that were common in the two centrality list were identified as central elements. K; degree, BC; Betweenness centrality, and CC; Closeness centrality. The asterisked genes are common between three groups of central nodes

**Row**	**Name**	**Description**	**K**	**BC**	**CC**
**1**	Htr1a*	5-hydroxytryptamine (serotonin) receptor 1A	72	0.23	0.62
**2**	Sstr2*	somatostatin receptor 2	70	0.10	0.59
**3**	Drd2*	dopamine receptor D2	70	0.13	0.61
**4**	Htr1b*	5-hydroxytryptamine (serotonin) receptor 1B	70	0.10	0.60
**5**	Penk	Preproenkephalin	69	0.00	0.59
**6**	Pomc	pro-opiomelanocortin-alpha	69	0.00	0.59
**7**	Oprm1	opioid receptor, mu 1	69	0.00	0.59
**8**	Npy	neuropeptide Y	69	0.00	0.59
**9**	Sst	Somatostatin	69	0.00	0.59
**10**	Chrm2*	cholinergic receptor, muscarinic 2	68	1.00	0.66

## Conclusion

In conclusion, this network analysis may provide additional clues for ibuprofen as a useful therapeutic agent in AD. The findings revealed that expression level of several genes that are involved in AD are changed by ibuprofen treatment. It may be a useful glance in the future investigation about the effect of ibuprofen in AD treatment and management.
